# AI Algorithms for Modeling the Risk, Progression, and Treatment of Sepsis, Including Early-Onset Sepsis—A Systematic Review

**DOI:** 10.3390/jcm13195959

**Published:** 2024-10-07

**Authors:** Karolina Tądel, Andrzej Dudek, Iwona Bil-Lula

**Affiliations:** 1 Department of Medical Laboratory Diagnostics, Faculty of Pharmacy, Wroclaw Medical University, 211 Borowska Street, 50-556 Wroclaw, Poland; iwona.bil-lula@umed.wroc.pl; 2 Institute of Mother and Child, 17a Kasprzaka Street, 01-211 Warsaw, Poland; 3 Department of Econometrics and Informatics, Faculty of Economics and Finance, Wroclaw University of Economics, Nowowiejska Street, 58-500 Jelenia Góra, Poland; andrzej.dudek@ue.wroc.pl

**Keywords:** artificial intelligence, machine learning, sepsis, neonatal, risk, progression

## Abstract

Sepsis remains a significant contributor to neonatal mortality worldwide. However, the nonspecific nature of sepsis symptoms in neonates often leads to the necessity of empirical treatment, placing a burden of ineffective treatment on patients. Furthermore, the global challenge of antimicrobial resistance is exacerbating the situation. Artificial intelligence (AI) is transforming medical practice and in hospital settings. AI shows great potential for assessing sepsis risk and devising optimal treatment strategies. **Background/Objectives:** This review aims to investigate the application of AI in the detection and management of neonatal sepsis. **Methods:** A systematic literature review (SLR) evaluating AI methods in modeling and classifying sepsis between 1 January 2014, and 1 January 2024, was conducted. PubMed, Scopus, Cochrane, and Web of Science were systematically searched for English-language studies focusing on neonatal sepsis. **Results:** The analyzed studies predominantly utilized retrospective electronic medical record (EMR) data to develop, validate, and test AI models to predict sepsis occurrence and relevant parameters. Key predictors included low gestational age, low birth weight, high results of C-reactive protein and white blood cell counts, and tachycardia and respiratory failure. Machine learning models such as logistic regression, random forest, K-nearest neighbor (KNN), support vector machine (SVM), and XGBoost demonstrated effectiveness in this context. **Conclusions:** The summarized results of this review highlight the great promise of AI as a clinical decision support system for diagnostics, risk assessment, and personalized therapy selection in managing neonatal sepsis.

## 1. Introduction

In recent decades, there has been a significant surge in the advancement of information technology. Alongside various sectors of business and industry, medicine has emerged as a notable participant and stakeholder in technological advancements, particularly in the domain of artificial intelligence (AI). Among the initial pivotal shifts, alongside advancements in diagnostic technologies, was the widespread adoption of electronic health records (EHRs) integrated within hospital information systems (HISs). This trend began in 2012, and projections indicate that by the start of 2022, electronic documentation had been implemented in approximately 90% of specialist medical offices in many countries. This signifies a substantial increase from the previous year, when only 50% had adopted electronic documentation by 2021 [1].

AI, a prominent domain within computer science, has undergone rapid implementation across a wide range of medical applications. Scientists and researchers are intrigued by its potential to emulate human cognitive functions for analyzing and recognizing patterns from data. It has its most valuable applications in drawing conclusions and decision-making based on, and generating, new evidence. [2]. Consequently, there is a growing interest in an AI-driven revolution in medicine. Already, various domains, particularly those dealing with vast datasets such as radiology, pathomorphology, genetics, and pharmaceuticals, have been at the forefront of AI implementation [3]. Furthermore, advancements in diagnostics in fields like cardiology and surgery represent highly innovative approaches. This is exemplified by the landmark event in 2018 when the FDA first authorized the use of AI algorithms to detect diabetic retinopathy in adults (IDx DR) [4].

In 21st-century medicine, there has been a significant increase in the volume of data generated throughout the diagnostic and therapeutic processes, estimated to comprise approximately 30% of the world’s total data volume [5]. This accumulated information creates a digital footprint similar to a patient’s digital twin, providing insights into each stage of the patient’s medical journey. Regrettably, the processing of these data is increasingly taxing for human medical personnel. Nonetheless, this abundance of data represents an untapped resource that could substantially augment medicine’s capacity to uncover new insights and correlations [6]. Hence, systematically collected data, organized within databases, offer invaluable opportunities for analysis, inference, comparison, and the construction of predictive models leveraging artificial intelligence. In the USA, medicine is already reaping the benefits of such solutions, with 171 of them having obtained approval as medical devices from the FDA by 2023 [7], providing tangible value to both patients and physicians.

### 1.1. AI in Medicine

The potential of AI is paramount to addressing healthcare resource shortages and enhancing global accessibility. Its applications, ranging from health monitoring to surgical treatment and the analysis of medical data, are pivotal in optimizing healthcare delivery [8]. Additionally, AI facilitates digital consultations and preventive screening, thereby improving access to care worldwide [9]. Recent studies have underscored the significance of computer-assisted diagnosis (CAD), particularly within hospital settings, where high sensitivity and specificity values enhance diagnostic accuracy. Furthermore, clinical decision support systems (CDSSs) represent another compelling application of AI. These computerized expert tools integrate individual patient characteristics to offer personalized analyses, specific recommendations, and crucial guidance for decision-making. They are gaining prominence for their utility in clinical diagnosis, pharmacotherapy, treatment selection, and predicting outcomes [10]. CDSSs exert a significant influence on the quality of clinical practice by improving accuracy in diagnosis and treatment through the personalized and timely dissemination of patient information [11]. Anchored in established guidelines, exemplars, and embedded rules, artificial intelligence (AI), particularly machine learning, demonstrates high efficacy in decision-making processes within healthcare settings [12].

### 1.2. Sepsis

The efficiency and aptitude of machines in the medical domain prompt an exploration of the optimal areas for their application. Particularly well suited are domains that necessitate swift responses based on a large volume of data and variables. In this study, we focus on addressing the challenge of sepsis and early neonatal sepsis. Sepsis and early-onset sepsis (EOS) remain a prevalent and severe concern for newborns, particularly preterm infants, where group B streptococcus (GBS) transmitted from the birth mother constitutes the most prevalent etiological agent, or Escherichia coli accounts for the highest mortality rates in this demographic. EOS within the first three days of life stands as the primary cause of infant morbidity and mortality [13,14]. Neonatal sepsis remains the foremost cause of neonatal mortality, resulting in over half a million deaths annually [15] and its incidence is 20 times higher in those born prematurely [16]. The diagnosis of neonatal sepsis presents significant challenges due to the rapid onset of symptoms and the necessity to integrate clinical indicators, inflammatory markers, and blood cultures promptly. These diagnostic complexities often require empirical treatment, typically with ampicillin and gentamicin or ampicillin and cefotaxime [17,18]. Several studies are underway to reduce the reliance on antibiotic therapy in neonates, given its numerous complications.

### 1.3. Objectives

In the pursuit of more effective disease management strategies, machine learning systems are being developed to monitor patient data from birth. These systems utilize algorithms to analyze medical histories alongside current symptoms, thereby identifying individuals at risk of sepsis and recommending protocols for management and treatment.

The following are the primary motivations for scientific interest in publications:

1. To review the research related to AI models for clinical decision support systems (CDSS) in neonatal sepsis, particularly early-onset sepsis (EOS).
2. To assess the importance of medical data inputs utilized in the construction of CDSSs, including their potential to develop trustworthy models for medicine.
3. To explore the feasibility of modeling the most effective antibiotic therapy through medical data analysis in sepsis to reduce empiric or untargeted antimicrobial treatment.
4. To evaluate the models employed by CDSSs and their effectiveness in classifying and predicting neonatal sepsis over time.

Furthermore, in our paper, we conduct a comparative analysis of publications alongside the clinical data underlying the aforementioned CDSSs, assessing their efficacy. Drawing from the insights gleaned from our review, we offer conclusions, recommendations, and limitations for further actions pertinent to the integration of such tools into routine clinical practice, emphasizing their potential impact on neonatal care.

## 2. Materials and Methods

### 2.1. Research Strategy

**Figure 1:**
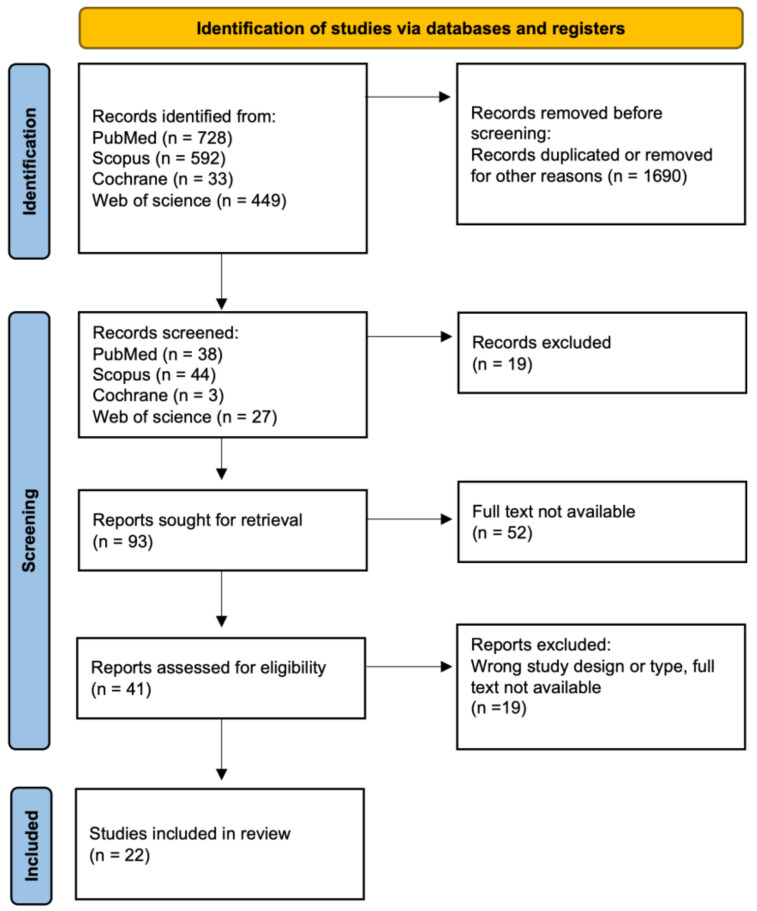
Flowchart for selection of publications.

Our approach involved a systematic literature review, encompassing various types of articles, including original research or reviews, written in English and published in English-language journals. This review adhered to the formal protocol in Figure 1 and followed the guidelines of Preferred Reporting Items for Systematic Review and Meta-Analyses (PRISMA) [19]. This study was not registered in the PROSPERO registry. The study selection process involved a comprehensive literature search, followed by three rounds of meticulous screening and filtering. Initially, articles published within the last decade were gathered, and duplicate entries were subsequently removed. Next, narrowing down to subsequent keywords was employed, and entire texts were analyzed to reach the final stage, resulting in 22 papers.

### 2.2. Inclusion Criteria

Systematic literature reviews (SLRs) focusing on the application of artificial intelligence in sepsis management within the healthcare context, irrespective of geographic location, were eligible for inclusion. Only publications available in English and published in peer-reviewed journals between January 1, 2014, and January 1, 2024, were considered. Searches were executed across multiple databases, namely PubMed, Scopus, Web of Science, and the Cochrane Library. The search strategy employed keywords including “artificial intelligence,” and the acronym “AI,” “sepsis,” and “neonatal,” combined with the logical operator “AND,” alongside wildcards, in the titles and abstracts of retrieved studies. Additional relevant terms, such as EOS, risk, prediction, and treatment, were also incorporated. Excluded from consideration were studies in the form of clinical trials and animal experiments, and those related to the exploration of experimental novel therapeutic modalities or diagnostic molecules determined by machine learning.

### 2.3. Data Extraction

All selected articles were thoroughly read, researched, and classified according to their primary categories. Subsequently, they were archived as Excel and Microsoft Word files (containing keywords, results, and conclusions) to streamline the filtering process.

The review was conducted by one of the authors following the outlined procedural steps:

1. Identification: Titles, keywords, and abstracts of all identified publications were scrutinized for alignment with the study’s objectives.
2. Verification of full texts: The complete texts of all publications identified in the preceding step were independently assessed for inclusion in the review and data extraction. This process adhered rigorously to the defined inclusion/exclusion criteria and study objectives.
3. Based on the collated materials, a presentation of the results was crafted, and conclusions with recommendations were formulated.

### 2.4. Research Questions

Formulating research questions is a critical step in defining the study’s objectives and expected outcomes. In light of this, we developed the following research questions to address the relevant issues identified through systematic analysis:

1. Are AI algorithms suitable for diagnosing sepsis as a support for medical professionals?
2. Can AI algorithms accurately assess the risk of sepsis and its rapid progression?
3. To what extent can AI algorithms contribute to enhancing treatment strategies for neonatal patients and decreasing antibiotic usage in the future?
4. What technological and clinical challenges arise in the utilization of AI for assessing sepsis progression and treatment, including early-onset sepsis (EOS)?

## 3. Results

### 3.1. Study Characteristics

A total of 1802 SLRs were identified. Based on the title and abstract reviews, 1690 hits were removed during the identification phase. The screening phase excluded 90 publications. After full-text analysis, 22 articles were finally included in the review (Figure 1) [20,21,22,23,24,25,26,27,28,29,30,31,32,33,34,35,36,37,38,39,40,41].

**Figure 2:**
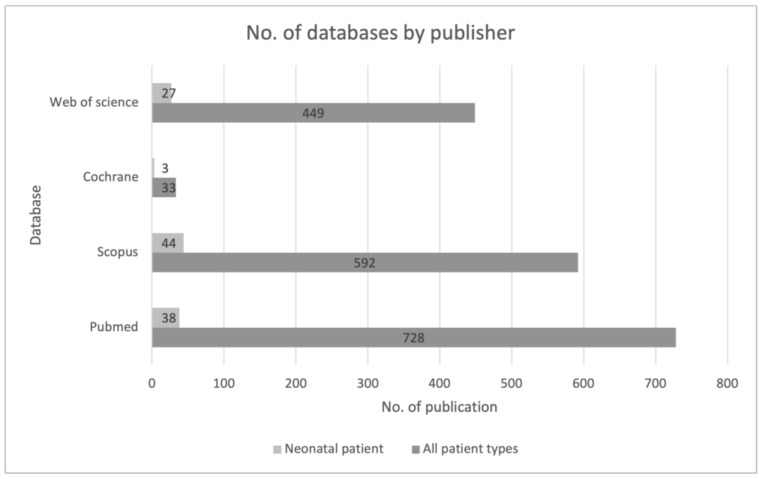
Review of sources of data used across included studies from the first stage of the identification process.

The largest searching database was the PubMed website, followed by Scopus. The first identification was based on the keywords and their synonyms, “artificial intelligence,” and the acronym “AI” AND “sepsis”. The search was then narrowed to the keyword “neonatal” and related synonyms. This allowed us to significantly narrow down the list of publications. It also shows how low and insufficient the number of publications regarding this youngest patient population is (Figure 2).

**Figure 3:**
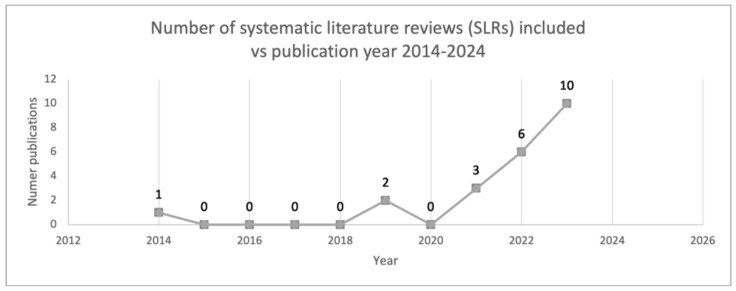
Number of SLRs included over the years.

The analyzed period spanned a decade. In the screening stage, it was observed that approximately 62% of articles were published between 2022 and 2023, with none originating in 2016. Ultimately, twenty-two articles were included in the analysis phase, among which ten were published in 2023 [20,23,25,27,28,29,30,31,32,35,39], accounting for 45% of the total. Notably, one of the earliest publications dates back to 2014 [20], marking a pioneering contribution to the field of AI and sepsis (Figure 3).

The primary country of origin for the research is the US, with additional representation from the Netherlands, Sweden, and Switzerland. Furthermore, studies from Asia and India are also present. The largest studies included 2519 and 2900 patients, and the smallest included 50. The scope of the review conducted is constrained, as only studies published in English were considered. It is conceivable that additional studies on the utilization of AI in sepsis risk assessment and treatment exist but were excluded based on the predetermined criteria.

### 3.2. Quality Assessment

The majority of studies in this field concentrate on developing models capable of promptly predicting the onset of neonatal sepsis or accurately categorizing patients as negative while awaiting confirmation from blood microbiological tests, integrating risk assessment over time [23,26,27,33,34,37]. However, limited attention has been directed towards predicting mortality [35,37] and customizing antibiotic therapy accordingly [24,32]. These studies evaluate the impact of biomarkers on therapeutic strategies, thereby identifying robust predictors for specific groups of antibiotics.

In terms of data types, information is gathered from medical records, medical device readings, and laboratory tests. Vital demographic data such as birth weight, gestational age, gender, delivery mode, prenatal and perinatal history, and comorbidities are collected. Key risk factors strongly associated with neonatal sepsis, including low birth weight, prematurity, perinatal history, premature rupture of membranes (PROM), C-reactive protein (CRP), procalcitonin (PCT), white blood cell count (WBC), platelet count (PLT), and respiratory or circulatory difficulties, are commonly included in these studies. However, the most consistent prognostic factors identified across the studies are low gestational age, CRP levels, WBC count, heart rate, and respiratory rate. The summary includes advanced characterization of the most significant papers, facilitating the presentation of results (Table 1). Neonatal movements are also considered significant in assessments, with one study focusing on their correlation with clinical parameters. Similar investigations have been conducted based on appearance, grimace, and crying sounds [25]. While promising results have been obtained, movement analysis has not achieved a satisfactory positive predictive value (PPV), with NPV (0.997–0.999) and PPV (0.009–0.022) [30]. Authors attribute this to the necessity of collecting a substantially larger dataset compared to studies analyzing only clinical parameters. Commonly utilized models include logistic regression, random forest, K-nearest neighbor (KNN), support vector machine (SVM), and XGBoost [21,23,37]. Cross-validation is employed to validate machine learning (ML) models, with many studies presenting receiver operating characteristic (ROC) curves and/or sensitivity and specificity to evaluate performance. This comprehensive evaluation is crucial, as a high AUC value does not always correspond to high sensitivity and specificity, which are vital for thorough evaluation and interpretation.

Following model evaluation, parameters strongly correlated with sepsis prediction or high risk of occurrence were selected. These include CRP, WBC, PLT, low gestational age, PROM, low birth weight, heart rate, respiratory support, and the APGAR score at 1–3–5–10 min. Additionally, advanced hematological, respiratory, and cardiovascular parameters have been evaluated and shown to perform well in predicting infection exacerbation. An example of a single patient with sepsis showed that the risk score could warn of sepsis up to 24 h earlier than current clinical practice [25]. Several studies have also investigated the application of AI in early-onset sepsis (EOS). The biomarker CRP notably influences the prediction of culture-proven EOS, and the second biomarker that had the greatest impact on prediction was WBC count. The potential of artificial intelligence and machine learning models in medicine is highly promising [36]. One study even devised an interactive sepsis early warning dashboard. Comparative analyses demonstrated high model performance, indicating that approximately 47% of patients could potentially be identified earlier. Such evidence strongly advocates for the utilization of AI to enhance and expedite clinical decisions in the early detection of sepsis [27].

## 4. Discussion

### 4.1. Strengths

Newborns, especially those in neonatal intensive care units (NICUs), face significant challenges, including invasive procedures, intensive treatments, and high mortality rates, with sepsis being a particularly threatening cause. The nonspecific nature of symptoms, rapid progression of infection, and prolonged wait for microbiological blood culture results present obstacles to appropriate care. Additionally, there is a disturbing trend in the overuse of antibiotic therapies, which is contributing to increasing antimicrobial resistance. Our review focused on analyzing reports concerning the prediction and classification of neonatal sepsis. We specifically examined the efficacy of artificial intelligence (AI) algorithms in clinical practice. The analysis demonstrated that machine learning exhibits high efficiency and can effectively complement or support the diagnostic process. Most studies were conducted in single centers, with only a few in international settings. In research, dominant countries are high-income countries, like the USA and EU members, with limited representation from Asia or India. The patient population’s characteristics, primarily its size, are crucial determinants of study success and result quality. A substantial representation of clinically and diagnostically confirmed sepsis cases is paramount, but challenging to attain. The authors consistently emphasize the need for larger, more comprehensive datasets in subsequent research efforts. Despite widespread adoption of electronic data collection methods, incomplete documentation remains a major limitation, leading to gaps in clinical and laboratory information. This inconsistency poses a significant threat to AI algorithms, as they rely on patterns learned from training data. The variability in disease progression and diagnostic procedures among patients further complicates algorithm development. Among the various models assessed, logistic regression, random forest, K-nearest neighbor (KNN), support vector machine (SVM), and XGBoost models were most frequently used and highly regarded. Key parameters for assessing and predicting sepsis in NICUs patients include low birth weight, prematurity, perinatal history, premature rupture of membranes (PROM), C-reactive protein (CRP), white blood cell count (WBC), platelet count (PLT), heart rate, and respiratory rate and function. However, research combining this information with optimal treatment pathways, including antimicrobial use, remains limited. While attempts have been made to select the most effective methods, further investigation is needed.

### 4.2. Limitations

Typical limitations observed in studies include missing or incomplete data, which affects the quality of artificial intelligence algorithms. Although data gaps usually do not result in the exclusion of patient data, they are recognized as potential limitations. This serves as a guideline for future researchers designing models. Additionally, the low number of positive sepsis cases (0.3–2 per 1000 live births [42]) contributes to a narrow patient population. This results in a naturally limited baseline for research or the need to gather data from many years or multiple centers, as such data are critical for building artificial intelligence algorithms. Furthermore, the diverse nature of sepsis, heterogeneous course, and age of onset (such as EOS, early-onset sepsis within the first 3 days of life, or LOS, late-onset sepsis) further necessitate the need for further detailed studies, particularly in the field of early-onset sepsis (EOS). Despite these challenges, machine learning has proven useful as a clinical decision support system for diagnostics, risk assessment, and personalized therapy selection in the management of neonatal sepsis.

The above study also has several limitations. First, our analysis was restricted to reviews based on selected keywords; consequently, there is a limitation that some publications that had nonspecific keywords may not have been included in the analysis. Second, we restricted the review to studies reported in English, so there is a risk that we excluded some publications from the analysis during this selection. We also cannot 100% rule out that some cited publications showed bias by not reporting on the unsatisfactory use of AI in neonatal sepsis. This is an extremely infrequent occurrence but as a cumulative effect may have overestimated the technological possibilities in this clinical area.

## 5. Conclusions

This review, focusing on the utilization of AI algorithms in modeling the risk, progression, and treatment of sepsis, including EOS, highlights their potential in modern medicine and clinical support. It underscores that the autonomy of doctors’ decisions remains safe, with attempts to implement systems based on automatic data analysis aimed at supporting rather than interfering with this process. Additional solutions are being sought, leveraging high computing power and real-time data comparison to provide supplementary information for medical personnel, akin to the advancements witnessed in imaging or laboratory diagnostics in years past. The major advantage of the analyzed AI models is utilizing information gathered during routine medical visits (clinical parameters) or laboratory results. There is no need for additional specialized tests, which could incur additional costs for hospitals.

Despite the challenges and barriers inherent in collecting and analyzing data in the neonatal population, AI algorithms have demonstrated promise in predicting and classifying sepsis cases and treatments. These challenges and limitations serve as a call for further research and analysis, necessitating a systemic approach to ensure data completeness, quality, and system interoperability, while also preparing medical staff to integrate new tools and recommendations effectively. Of particular interest is whether the insights provided by new algorithms have the potential to revise current standards or guidelines for managing neonatal sepsis, especially in terms of diagnosis and treatment criteria, an area where multiple perspectives indicate the potential contribution of AI. An intriguing avenue for exploration lies in assessing the extent to which empiric antibiotic therapy use in the pediatric population can be reduced, and whether AI can offer novel evidence in this regard. However, the development of effective AI models requires training on diverse datasets, necessitating research beyond individual regions or countries. Therefore, international collaboration remains crucial in deepening the understanding and application of AI in medicine, including sepsis diagnostics, to enhance the efficacy of such tools.

Ethical considerations and concerns regarding over-reliance on device-generated results are frequently discussed within the context of AI in medicine. There is a need to verify and evaluate content provided by automated methods, comparing it with human assessments for comprehensive evaluation. This holistic approach to AI in medicine seeks not to diminish the role of humans, but rather to complement and enhance everyday work, ensuring higher quality and safety standards. The underlying interest and motivation in advancing the application of artificial intelligence in clinical practice are rooted in improving medical practices and enhancing patient treatment outcomes, offering a promising prospect for the future.

## Figures and Tables

**Table 1:** Characteristics of selected studies included in the review of the use of AI in the assessment of neonatal sepsis.

Title	Author	Year	Country	Number of Patients in the Study	AI and Statistic Models	Scope of Research	Top Significant Parameters for the Models
Medical decision support using machine learning for early detection of late-onset neonatal sepsis	Subramani Mani et al. [24]	2014	USA	299 (90 positive)	Support Vector Machine (SVM), the naive Bayes (NB) classifier, tree augmented naive Bayes (TAN), averaged one dependence estimators (AODE), K-nearest neighbor (KNN), classification and regression trees (CART), andom forests (RF), logistic regression (LR), lazy Bayesian rules (LBR)	sepsis, antibiotic treatment	chorioamnionitis, white blood cells count, anemia of prematurity, maternal age and resuscitation at birth to be strong predictive factors for LOS
Machine learning models for early sepsis recognition in the neonatal intensive care unit using readily available electronic health record data	Aaron J. Masino et al. [22]	2019	USA	1188 (375 positive)	logistic regression (LR), naïve Bayes (NB), SVM with a radial basis function kernel, K-nearest neighbors (KNN), Gaussian process, RF, AdaBoost, and gradient boosting	sepsis, antibiotic treatment	heart rate difference, systemic and diastolic blood pressure, platelet count, I/T ratio, mean arterial pressure, gestational age
Optimizing neural networks for medical data sets: A case study on neonatal apnea prediction	Rudresh Deepak Shirwaikar et al. [41]	2019	India	367	KNN, SVM, RF, MPL decision trees, deep autoencoders	sepsis	birth weight, heart rate, desaturation, gestation age
Aiding clinical assessment of neonatal sepsis using hematological analyzer data with machine learning techniques	Huang, B et al. [21]	2021	USA	2900 (2032 sepsis positive)	extreme gradient boosting (XGB), Random Forest (RF), and Support Vector Machine (SVM)	sepsis	neutrophil fluorescence intensity (NE_SFL), neutrophil cell size (NE_FSC), neutrophil dispersion width (NE_WY), gestational age, and monocyte fluorescence intensity (MO_Y)
Machine Learning Approaches to Predict In-Hospital Mortality among Neonates with Clinically Suspected Sepsis in the Neonatal Intensive Care Unit	Jen-Fu Hsu et al. [37]	2021	Taiwan	2472 (1095 posotove)	DNN model, k-nearest neighbors (k-NN), vector machine (SVM), random forest (RF), extreme gradient boost (XGB), Glmnet, regression tree algorithm (Treebag)	sepsis	requirement of ventilator support, feeding conditions intravascular volume expansion
Machine Learning Used to Compare the Diagnostic Accuracy of Risk Factors, Clinical Signs and Biomarkers and to Develop a New Prediction Model for Neonatal Early-onset Sepsis	Stocker, Martin et al. [26]	2022	Netherlands, Canada, Czech Republic and Switzerland	1685 (28 positive)	CSs, Random Forest (RF)	early-onset sepsis (EOS)	C-reactive protein, leukocyte count, platelet count, birth weight, gestational age
A Continuous Late-Onset Sepsis Prediction Algorithm for Preterm Infants Using Multi-Channel Physiological Signals From a Patient Monitor	Zheng Peng et al. [30]	2022	Netherlands	127	extreme gradient boosting (XGB), k-nearest neighbors (KNN), logistic regression (LR), support vector machine (SVM)	LOS	HRV features, breathing features, movement features, combination of HRV and breathing features and combination of all features
Newborn Cry-Based Diagnostic System to Distinguish between Sepsis and Respiratory Distress Syndrome Using Combined Acoustic Features	Zahra Khalilzad et al. [32]	2022	Canada, Lebanon	50 (17 positive)	deep feedforward neural network (DFNN), vector machine (SVM) model, convolutional neural network (CNN), Multilayer Perceptron (MLP)	sepsis	recording newborn cry-based
The use of artificial intelligence in the diagnosis of neonatal sepsis	Dž. Gojak et al. [33]	2022	Bosnia and Herzegovina	1000	artificial neural network (ANN)	sepsis	Body temperature, C-reactive protein, leukocyte count, Platelet count
Neonatal Disease Prediction Using Machine Learning Techniques	Yohanes Gutema Robi et al. [23]	2023	Etiopia	180	XGBoost (XGB), Random Forest (RF), and Support Vector Machine (SVM)	late-onset sepsis (LOS)	APGAR, C-reactive protein, resuscitation, low lung volume and bleaching (LLVB), intercostal subcostal retractions (ICSCR), SpO2, gestational age, white blodd cells count, convulsions, respiratory function, weight
Vital sign-based detection of sepsis in neonates using machine learning	Antoine Honoré et al. [25]	2023	Sweden	325 (20 positive)	model Hidden Markowa	sepsis	birth weight, gender and postnatal age, heart rate, respiratory characteristics
Development and clinical impact assessment of a machine-learning model for early prediction of late-onset sepsis	Merel (A.M.) van den Berg et al. [27]	2023	Netherlands	2519 (389 positive)	logistic regression (LR), GAM i XGBoost	LOS	C-reactive protein, leukocyte count, neutrophil count and thrombocyte counts
Prediction of mortality among neonates with sepsis in the neonatal intensive care unit: A machine learning approach	Colleen O’Sullivan et al. [31]	2023	India	388 (184 positive)	naive bayes (base line model), ogistic regression, sequential minimal optimization (SMO), classification and regression tree (CART), Random Forest	EOS, LOS	PROM, absent end diastolic flow, chorioamnionitis-maternal predictors; preterm birth, birthweight (>2500 g), appearance, pulse, grimace, activity, and respiration, APGAR at 5 min, C-reactive protein

## Data Availability

Not applicable.
